# Sulphate of Magnesia—Abstract

**Published:** 1899-12

**Authors:** J. C. Culbertson

**Affiliations:** Cincinnati, Ohio


### THE DENTAL REGISTER.

VOL. LIII.] DECEMBER, 1899. [No. 12.

### COMMUNICATIONS.

### Sulphate of Magnesia—Abstract.

BY J. C. CULBERTSON, M.D., CINCINNATI, OHIO.

In the blaze and glory of the heraldry of new remedies and elegant pharmacy, physicians are apt to forget, or at least overlook, some of the old, tried and true preparations. In diverting attention to one of these, no intention is made to cast any slur on, or to detract from, the value and usefulness of others.

Half a century ago Pereira proclaimed that sulphate of magnesia was by far the most commonly employed purgative, both by the public and the medical profession, and to state all the cases in which it is administered would be to enumerate nearly the whole catalogue of known diseases.

Following in the same line of observation, Stille says it is taken incomparably oftener without a physician's advice than with it, referring to it as a certain, safe and mild purgative. Similar observations have been made by all writers on our materia medica and I wish to echo their expressions, and say that I believe sulphate of magnesia is the most useful remedy in the long list of medicinal preparations at our command. In the beginning of any treatment of any disease, to clear the alimentary canal of any irritant matter which may be present, is nearly always called for, and to accomplish this purpose without weakening or complicating the case, there is no one remedy so effective.

Notwithstanding all that has been written and stated so favorably as to the use of this potent remedy, I am inclined to believe that it is not yet fully appreciated for its many virtues. It may seem strange and even presumptuous to say this, in view of all that has been said, and in the face of the fact that sulphate of magnesia has become a universal domestic remedy. However, as used by the people, it is only as a simple, safe purgative that it finds place in every-day family use. In this rank it holds precedence all the time. As a purgative, its most common administration is in the morning, and in a dose of from half an ounce to an ounce. This may be greatly improved on, by giving half a dram to a dram, administered in a pint of water. The mechanical effects of the water are beneficial and aid in establishing increased peristaltic action of the bowels. The remedy, when given in this manner, is peculiarly cooling; it is refrigerative. The same dose given at bedtime rarely acts even as a laxative, but becomes a most excellent diuretic, and also stimulates the skin so as to literally rid the body of accumulations of detritus, wherever it may be located. This is a property possessed in a high degree by sulphate of magnesia. In fact, it may be placed at the very head of the column of diuretics, that are certain, safe and mild in action.

For a cure of dysentery magnesia sulphas comes nearer being a specific than any remedy at our command. In giving it, in this disease, from a dram to an ounce may be used. If large doses are given the discharges are apt to be stopped too abruptly; a dram is better than an ounce and may be repeated every hour or two.

For relief and cure of sick headache, it should be given in half-dram to dram doses every morning, dissolved in a goblet of water. Equally good results or even better may be obtained by administering at bedtime. The goblet or pint of water should be insisted on with every dose. The temperature of the water may be that which is most agreeable to the patient, although I have thought warm water, say 100 degrees, more potent than that at a lower temperature.

In cases of hemorrhoids and all congested conditions of the rectum and other pelvic organs, sulphate of magnesia is almost a specific in a large number of instances; where the patient is troubled with flatulence, an addition of peppermint to the salt solution is very effective in affording relief.

In gynecologic cases, and particularly in peritonitis, sulphate of magnesia is again found at the top of the column of useful remedies. As a cholagogue it is again in the front rank, but it is useless and time-absorbing to refer to special indications for this old stand-by. However, especial attention is directed to its value as a diuretic when given at night on retiring. The usefulness of so-called small doses in large draughts of water, preferably warm, is insisted on.

One word more. Physicians writing prescriptions for this remedy should always write for sulphate of magnesia, and should absolutely abandon the words "Epsom salts." They should not tell patients to take a specific amount of the drug, but should carefully write prescriptions for it, directing some carminative with it. Let the dictum go forth that "salts" is a meaningless name applicable to several chemicals, and is not especially significant of sulphate of magnesia; that "Epsom" is the name of a town, and not an appendage of a therapeutic compound. Get rid of the domestic names for this invaluable medicine, and do it at once, by never permitting ourselves to use them, and thus retract what should never have been given to the people.

In this relation I take the liberty of stating that there has been entirely too much giving of our knowledge of remedies to the people, and it actually takes from them much of their specific value. Very many lives have been sacrificed at the altar of dangerous remedies in the hands of the laity, and the people should be educated to believe that disease in every form is a state for pathologic study, and that the simplest as well as the most complex remedies in our materia medica are potent for harm as well as for good when in the hands of those not qualified to administer. These things are the secrets of our profession. They are essentially our bread and butter, they are our living, and everything, and should be dignified by our own treatment of them.

I do not presume to tell you much of anything that is new in regard to an old and useful remedy, but rather make an appeal to take this therapeutic agent out of its present position as a domestic remedy and place it where it justly belongs, in the category of therapeutic agents of the greatest value and respectability.

